# Glycosylated proteins in the protozoan alga *Euglena gracilis*: a proteomic approach

**DOI:** 10.1093/femsle/fnac120

**Published:** 2022-12-07

**Authors:** Ellis C O’Neill

**Affiliations:** School of Chemistry, University Park, Nottingham NG7 2RD, United Kingdom

**Keywords:** alga, gracilis, Euglena, glycoproteins, glycans

## Abstract

Protein glycosylation, and in particular *N*-linked glycans, is a hallmark of eukaryotic cells and has been well-studied in mammalian cells and parasites. However, little research has been conducted to investigate the conservation and variation of protein glycosylation pathways in other eukaryotic organisms. *Euglena gracilis* is an industrially important microalga, used in the production of biofuels and nutritional supplements. It is evolutionarily highly divergent from green algae and more related to kinetoplastid pathogens. It was recently shown that *E. gracilis* possesses the machinery for producing a range of protein glycosylations and make simple *N*-glycans, but the modified proteins were not identified. This study identifies the glycosylated proteins, including transporters, extracellular proteases, and those involved in cell surface signalling. Notably, many of the most highly expressed and glycosylated proteins are not related to any known sequences and are, therefore, likely to be involved in important novel functions in Euglena.

## Teaser

Lectin affinity and proteomics identified glycosylated and extracellular proteins, including transporters, proteases and signalling components, in the evolutionarily and industrially important protozoan alga *Euglena gracilis*.

## Introduction

Euglena are a class of mixotrophic protozoa that live in predominantly freshwater aquatic environments (Buetow 1968). Most possess a green secondary plastid derived by endosymbiosis of a chlorophyte algae (Zakryś et al. 2017), and there have been at least four endosymbiotic genome transfers, as well as significant horizontal gene transfer, during their evolutionary history (Henze et al. 1995). Uniquely among plastid-containing cells, the chloroplast can be lost from photosynthetic Euglena without compromising their viability, due to duplication of all major pathways present in the chloroplast elsewhere in the cell (Inwongwan et al. 2019). Euglenids are related to the well-known kinetoplastid unicellular parasites *Trypanosoma* and *Leishmania*, as part of the phylum Euglenozoa (Adl et al. 2019). Euglena have been subject to scientific study for hundreds of years, but have recently become more intensely researched due to their considerable potential for biotechnological exploitation (Gissibl et al. 2019, Ebenezer et al. 2022).

*Euglena gracilis*, the most well-characterized member of this group, has been studied for the production of vitamins A, C, and E (Takeyama et al. 1997), essential amino acids, and polyunsaturated fatty acids (Korn 1964). The storage polysaccharide, paramylon (Rodríguez-Zavala et al. 2010), makes up to 85% of algal dry weight when grown aerobically in light, whilst under anaerobic conditions wax esters can make up over 50% of the dry weight (Inui et al. 1982). These high value components have led to *E. gracilis* being cultivated as a food supplement (Zeng et al. 2016).Recent work on the transcriptome and genome of *E. gracilis* has revealed the biosynthetic pathways for these valuable compounds (O’Neill et al. 2015a, Ebenezer et al. 2019).

Euglena have been reported to have complex carbohydrates bound to their surface (Barras and Stone 1965, Bouck et al. 1978) and lectin- and antibody-based profiling revealed a complex glycan surface, with some similarities to plant galactans and xylans (O’Neill et al. 2017). There are a wide range of carbohydrate active enzymes in the *E. gracilis* transcriptome, implying a capability for the synthesis of complex carbohydrates (O’Neill et al. 2015b), and the cells contain a wide range of the sugar nucleotides needed as substrates for the synthesis of these polysaccharides (O’Neill et al. 2017). The exact nature of the complex surface carbohydrates in Euglena remains to be uncovered.

Protein glycosylation is a major post-translational modification in eukaryotic organisms, stabilizing surface proteins and providing specific intercellular interactions (Varki et al. 2017). *Euglena gracilis* expresses a range of enzymes necessary for the glycosylation of proteins: it has all of the genes necessary for the biosynthesis of GPI anchors, which anchor proteins into the phospholipid bilayer via a sugar–lipid tag, including the key transamidase for attaching the protein (O’Neill et al. 2015a); there are three members of the GT41 family of glycosyltransferases, which transfer *N*-acetylglucosamine to serine and threonine residues of proteins in the cytosol (O’Neill et al. 2015b); *N*-acetylglucosamine-1-phosphate transferase activity has been detected in membrane preparations of *E. gracilis* cells (Ivanova et al. 2017), likely involved in modifying proteins to target them to different subcellular compartments; and sequences for all of the enzymes required for the synthesis of the highly conserved *N*-glycan precursor can be identified in the transcriptome, as well as three sequences for the transferases that transfer this preformed oligosaccharide to the target proteins (O’Neill et al. 2015a). Together, these results indicate that Euglena encodes the ability to form complex posttranslational glycosylation of proteins. Protein *N*-glycan profiling of *E. gracilis* revealed that there was indeed protein glycosylation, mostly with high mannose type glycans with a small proportion modified with aminoethylphosphonate (O’Neill et al. 2017). No evidence was found for complex *N*-glycans or for *O*-linked glycans on Euglena proteins and the proteins carrying these modifications were not identified.

This study uses lectin-mediated protein isolation and proteomic analysis to identify the proteins that are decorated with these glycans in order to understand the contribution of protein glycosylation to the Euglena proteome and inform future production of pharmaceutical proteins.

## Materials and methods

### Culturing

*Euglena gracilis* Z (CCAP1224/5Z) was grown in 15 ml of EG:JM + glucose (15 g l^−1^) at 30°C with shaking (50 rpm) and illumination (∼60 µmol m^2^ s^−1^) until late log phase (10 days) in triplicate. Cells were harvested by centrifugation (1000 ×*g*) and resuspended in supernatant (1 ml).

### Glycoprotein preparation

The resuspended Euglena cells from the culturing (1 ml) were diluted with 5x binding/wash buffer (0.25 ml) containing phenylmethylsulfonyl fluoride (2 mM) and lysed by sonication (3 × 10 s, 25% amplitude, 30 s off between each pulse) and centrifuged (5 min, 1000 × *g*). Not all cells were lysed. Total lysate containing the equivalent of 1.1 mg of protein (Easy Bradford BioRad, BSA standards) was then used for glycoprotein purification using both ConA and wheat germ agglutinin (WGA) Glycoprotein Isolation Kits (Thermo Scientific) according to the manufacturer’s instructions. Protein quality was assessed using silver-stained SDS-PAGE (Bolt 4%–12% Bis-TRIS plus, Invitrogen) using SeeBlue Plus2 Prestained Protein Standard (Thermo Fisher Scientific) as the standard.

### Protein digestion and analysis by mass spectrometry

Protein digestion and analysis was performed by the Advanced Proteomics Facility at Oxford University. Protein samples were digested according to the filter-aided sample preparation (FASP) procedure (Wiśniewski et al. 2009). Peptide digest was treated with PNGase F and analysed by nano-liquid chromatography tandem mass spectrometry (nano-LC/MS/MS) on an Orbitrap Elite™ Hybrid Ion Trap-Orbitrap Mass Spectrometer (Thermo Scientific) using collision-induced dissociation (CID) fragmentation.  Peptides were loaded on a C18 PepMap100 precolumn (300 µm i.d. × 5 mm, 100 Å, Thermo Fisher Scientific) at a flow rate of 12 μl min^−1^ in 100% buffer A [0.1% formic acid (FA) in water]. Peptides were then transferred to an in-house packed analytical column heated at 45°C (50 cm, 75 µm i.d. packed with ReproSil-Pur 120 C18-AQ, 1.9 µm, 120 Å, Dr. Maisch GmbH) and separated using a 60 min gradient from 8% to 30% buffer B [0.1% FA in acetonitrile (ACN)] at a flow rate of 200 nl min^−1^. Survey scans were acquired at 120 000 resolution to a scan range from 350 to 1500 m/z. The mass spectrometer was operated in a data-dependent mode to automatically switch between MS and MS/MS. The 10 most intense precursor ions were submitted to CID fragmentation using a precursor isolation width set to 1.5 Da and a normalized collision energy of 35. Database search was carried out using MaxQuant (1.6.3.4) against the nonredundant Euglena proteome available at https://jicbio.nbi.ac.uk/euglena/(O’Neill et al. 2015a), with default parameters and including deamidation on Asn residues as variable modification for *N*-glycosylation sites identification. The mass spectrometry proteomics data have been deposited to the ProteomeXchange Consortium via the PRIDE (Perez-Riverol et al. 2019) partner repository with the dataset identifier PXD030579.

### Extracellular proteins

The supernatant from the cell culture was filtered (0.2 µm) and lyophilized. The material was dissolved in ammonium bicarbonate (2.5 ml, 50 mM) and desalted using a PD 10 column (Amersham Pharmacia Biotech AG) equilibrated and eluted with ammonium bicarbonate (50 mM), the resultant material was again lyophilized and dissolved in MQ H_2_O (0.4 ml).

### Data analysis

All Total, ConA, and WGA samples were normalized with respect to an average of the 165 proteins detected in every sample. The Ext and Total samples were normalized to an average of the 81 proteins detected in both of these samples. Proteins that were differentially detected between the different treatments and the total proteome (*P* < .05, Student’s *t*-test, two tail) were included in further analysis. Using Blast2GO, protein sequences were matched to sequences in the NCBI nonredundant protein sequence database and assigned GO terms based on this. Sequences that returned no hits were then searched in the TriTrypDB, the most comprehensive datasets for relatives of Euglena, the kinetoplastid parasites, (Aslett et al. 2010).

## Results and discussion

Using standard proteomic techniques, the total proteome, glycan-containing proteome, and extracellular proteome were analysed from *E. gracilis* grown in a high yielding mixotrophic culture. It is notable that many of the most abundant proteins in all of the experimental samples in this study, as in previous work (Ebenezer et al. 2019), are not linked to known sequences using BLAST. Many of those that do have known related sequences cannot be associated with GO terms or predicted functions, and together this indicates that some of the most highly abundant proteins in *E. gracilis* have no known function. As the tools used to identify protein sequences have not been developed or optimized for use with Euglena, related proteins may not be successfully identified, and caution should be used when interpreting these results. It should also be noted that, due to limitations with the analytical techniques, the failure to detect a protein does not confirm its absence, but that it may not produce detectable peptides, be below the limit of detection or be masked by other, much more abundant, proteins.

The asparagine, which is glycosylated, can be identified by a mass deviation of 1 Da from the expected mass, caused by the cleavage of the *N*-glycan by PNGase-F treatment during the sample preparation. Peptides may not be detected in the modified form and the protein may be identified by other peptides, and so absence of this signal does not indicate absence of glycosylation of a protein. Only 88 of the 382 peptides annotated as containing this *N*-deamidation, appear to have the canonical NX(S/T) recognition signal for glycosylation. Many of the glycosylation sites detected in this work are associated with proteins that would not be expected to be targeted to the ER/Golgi and secreted, and thus would not be expected to be glycosylated. It should be noted that proteins targeted to the chloroplast in *E. gracilis* are initially targeted to the ER/Golgi (Záhonová et al. 2018), and thus would be exposed the protein glycosylation machinery. Chemical deamidation of asparagine can also occur, giving rise to false identification of glycosylation sites (Palmisano et al. 2012). The high ratio of noncanonical sites in this dataset raises serious concerns with the use in Euglena of protein glycosylation site-prediction tools that rely on this recognition motif and indicates that there may be some other signal to target glycosylation in this organism. There are three sequences for the GT66 oligosaccharyltransferases, which transfer the preformed glycan to the protein asparagine, encoded in the *E. gracilis* transcriptome (O’Neill et al. 2015a) and it is possible that these have different specificities. Further experiments would be required to validate the true glycosylation sites.

### Total proteins

**Figure 1.**
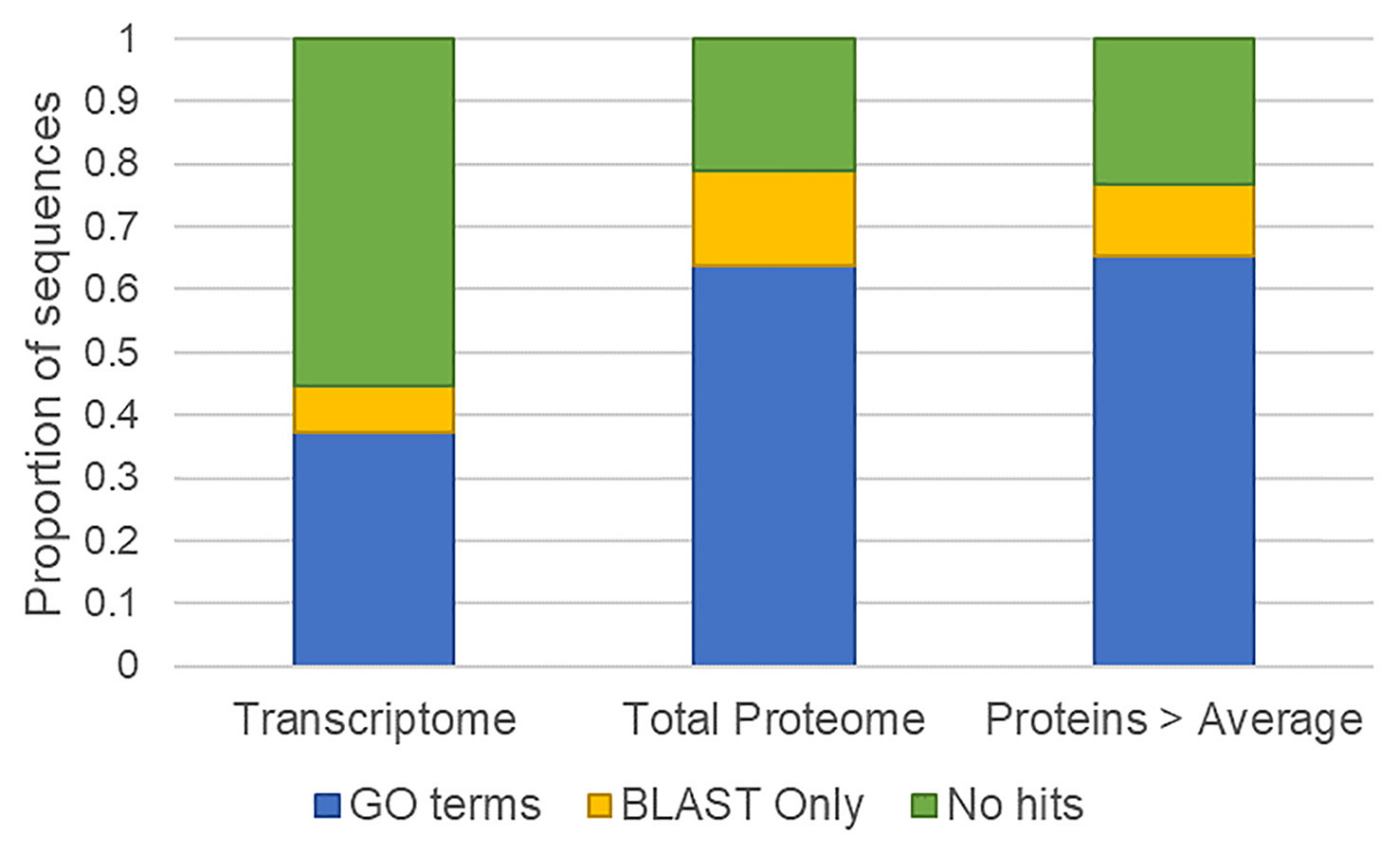
Euglena sequence identification. Proportion of sequences with identified GO terms and BLAST hits (blue), BLAST hits only (yellow), and neither (green) using Blast2GO in the *E. gracilis* transcriptome (O’Neill et al. 2015a), total proteome and proteins detected above average (this study).

A total of 1309 proteins were detected in all samples from the total proteome (see Supplementary Data), and of these 63% (836) have identified GO terms (see Fig. 1), much higher than the 37% of the total transcripts which have GO terms mapped (O’Neill et al. 2015a). Of the 130 proteins detected above the average, 30 do not have any BLAST hits, and 85 have GO terms identified. This indicates that the proteins that can be detected are more likely than those predicted from the transcriptome to have known related sequences, possibly indicating the many of the predicted but unknown proteins are produced at a lower level or the transcripts do not encode for translated proteins. However, there are still many proteins that are unique to Euglena that are produced at relatively high levels and would repay further study.

In order to identify the likely subcellular location of these abundant proteins, protein-targeting predictions were performed, using bioinformatic tools that have previously been used for Euglena proteins (Inwongwan et al. 2019). Protein transport into Euglena chloroplasts occurs first via the secretory pathway and the Golgi apparatus using a secretion signal, followed by targeting to the chloroplast using a plastid-targeting signal (Durnford and Gray 2006). Therefore, to confirm whether a protein was truly secreted or sent to the chloroplast, any predicted signal peptides were removed and the prediction repeated, revealing any masked plastid-targeting signal. TargetP (Almagro Armenteros et al. 2019) predicted that four of the 20 most abundant proteins are targeted to the mitochondria, two to the chloroplast, and one secreted, whilst WoLF PSORT (Horton et al. 2007) predicts six to be targeted to the chloroplast, four to the mitochondria, and one secreted (see Table 1). These results suggest the chloroplast and mitochondria contain some of the most abundant proteins in the cell.

**Table 1.** The 20 most abundant proteins in the total Euglena proteome.

Sequence name	Putative sequence description	Average intensity	SD	TargetP prediction^23^	WoLF PSORT prediction^24^
16406^+^	Glyceraldehyde 3-phosphate dehydrogenase	17.4	0.0367	Other	Cytosol
3371^+^	Stromal 70 kDa heat shock-related protein, chloroplastic-like	13.1	2.88	Chloroplast*	Chloroplast*
12260^+^	Phosphoglycerate kinase	9.85	2.14	Other	Cytosol
8709	Oxygen-evolving enhancer protein 1, chloroplastic	9.50	1.31	Chloroplast	Chloroplast
8430	Putative mitochondrial heat-shock protein hsp70	9.35	1.09	Other*	Cytosol*
6665^+^	Phosphopyruvate hydratase	8.76	0.566	Other*	Cytosol*
61646	60 s acidic ribosomal protein p2	7.99	0.290	Other*	Extracellular* (chloroplast)
4720	NA	7.73	1.19	Other	Mitochondria
25934^+^	Oxygen-evolving enhancer protein 3	7.42	1.11	Signal peptide (signal peptide)	Chloroplast
25617^+^	Electron transfer flavoprotein subunit alpha	6.64	1.38	Other*	Chloroplast*
8433	NA	6.52	0.535	Other	Nucleus
8912^+^	Putative ATPase beta subunit	6.51	1.13	Mitochondria*	Mitochondria*
12357	Elongation factor 1-alpha	5.91	0.663	Other*	Cytosol*
14010	Beta-tubulin	5.45	0.352	Other*	Nucleus*
7178^+^	Chaperonin HSP60, mitochondrial precursor	4.97	0.891	Mitochondria	Mitochondria
23135	NA	4.89	0.0541	Other*	Cytosol*
7258	Midasin^x^	4.75	0.335	Other	Nucleus
8679	Putative dihydrolipoamide dehydrogenase	4.42	0.969	Mitochondria	Chloroplast
5458^+^	Molecular chaperone DnaK	4.14	2.14	Mitochondria	Mitochondria
4275^+^	ATPase beta subunit	3.97	1.01	Other	Chloroplast

The full list is available in the supplementary file. NA are sequences with no homologues identified by BLAST. Any secretory signal peptides identified were removed and the analysis repeated, with results shown in brackets. ^+^ indicates deamidation sites that were detected. * indicates sequences that do not start with a M and so may be truncated sequences that do not contain the targeting sequence present in the protein. ^x^indicates homologues that could only be found in TriTrypDB.

### ConA glycoprotein isolation

Concanavalin A (ConA) is a protein that specifically binds mannose, such as is found in simple *N*-glycans, and glucose which can be found on the termini of *N*-glycans. Using an immobilized ConA column to enrich for *N*-glycan displaying proteins, a total of 86 proteins were detected at a significantly higher rate than in the total proteome, and 50 of these were not detected in the total proteome at all (see Table 2). A total of 37 of these ConA-enriched proteins had BLAST matches and 30 mapped to GO terms. In total, six of these are likely to be involved in signalling, three in sugar metabolism, two in transport, and there are four likely proteases. There are four proteins that are linked to biosynthesis, two to redox balance, and 12 involved in core housekeeping roles, which would expect to be cytosolic and thus not glycosylated. A total of 13 of the 86 proteins had an *N*-deamidation site detected in at least one of the samples. Of the proposed cytosolic housekeeping genes, this modification was noted in: 7967, a trypanothione reductase that has a deamidation site in all ConA samples, as well as the single WGA sample in which it was detected; 5325, a small nucleolar ribonucleoprotein U3, with one *N*-deamidation site in just one ConA sample; and 32750, a RNA scaffolding Sm-like protein, with deamidation in all WGA samples, although it was not detected significantly over the control in them, but not with no deamidation detected in any of the ConA samples. Only six of the ConA-enriched proteins were predicted to be secreted, again highlighting the limitations of predicting protein targeting in protozoa.

**Table 2.** The 10 most abundant proteins enriched in ConA glycoprotein isolation. The full list is available in the supplementary file.

Sequence name	Putative sequence description	Average intensity	SD	Ratio ConA/total	*P*-value	TargetP prediction^23^	WoLF PSORT prediction^24^
14865^+^	Predicted protein	33.0	12.0	35.4	0.0438	Chloroplast*	Chloroplast*
361	NA	7.87	2.32	7.99	0.0357	Other*	Nucleus*
17372^+^	Vitamin B6 biosynthesis protein	5.03	0.737	2.45	0.0124	Other	Cytoplasm
4137	Predicted protein^x^	2.76	0.606	13.9	0.0169	Other	Cytoplasm
53965	UV excision repair protein Rad23	1.61	0.309	5.77	0.0172	Other*	Cytoplasm*
15475^+^	GDP-mannose 4,6 dehydratase	0.96	0.202	3.30	0.0121	Other*	Cytoskeleton*
37872	NA	0.80	0.243	12.4	0.00344	Other*	Nucleus*
538	NA	0.76	0.284	103	0.044	Other	Nucleus
22038	Charged multivesicular body protein 4a	0.63	0.158	2.94	0.0355	Other	Nucleus
32626^+^	Transcription factor BTF3 homolog 4-like	0.63	0.200	***	0.0321	Other	Nucleus

NA are sequences with no homologues identified by BLAST. *** are proteins not detected in the total protein sample. ^+^ indicates deamidation sites that were detected. * indicates sequences that do not start with a M and so may be truncated sequences that do not contain the targeting sequence present in the protein. ^x^indicates homologues that could only be found in TriTrypDB.

### WGA glycoprotein isolation

WGA is a protein that specifically binds GlcNAc (or sialic acid, which is not present in Euglena; O’Neill et al. 2017), found in the core of *N*-glycans. A total of 675 proteins were detected in the sample eluted from the WGA glycoprotein isolation column. Of these, 16 were detected at a statistically significant rate higher than in the total cellular proteome (see Table 3), of which six were also detected in the ConA glycoprotein isolation sample. Just six of the 16 had matches in the nonredundant protein database and just four of these mapped to GO terms. These are a protein possibly involved in DNA repair, an oxidoreductase, a protein likely involved in retrograde signalling, and an integral membrane protease. It is possible that the WGA-enriched proteins also contain an *O*-GlcNAc residue, a cytosolic protein modification found in eukaryotes with a role in cellular signalling and nutrient response (Zeidan and Hart 2010).

**Table 3.** Proteins enriched in WGA glycoprotein isolation.

Sequence name	Putative sequence description	Average intensity	SD	Ratio WGA/total	*P*-value	TargetP prediction^23^	WoLF PSORT prediction^24^
361^#^	NA	5.61	0.180	5.70	0.000253	Other*	Nucleus*
24159	UV excision repair protein RAD23 homolog B	1.57	0.407	5.80	0.0276	Other*	Chloroplast*
9057^#^	NA	0.482	0.174	***	0.0409	Other	Nucleus
20266^#^	S-(hydroxymethyl) glutathione dehydrogenase/class III alcohol dehydrogenase	0.423	0.118	6.01	0.0131	Other*	Cytoplasm*
19374	NA	0.369	0.133	22.5	0.0485	Other*	Nucleus*
255	Kinesin-like protein KIF16B	0.188	0.0478	9.31	0.0267	Other	Cytoplasm
59^#^	NA	0.144	0.0487	4.49	0.0294	Other	Cytoplasm
12064	NA	0.136	0.0376	***	0.0245	Other*	Cytoplasm*
27863^#^	NA	0.109	0.00111	***	3.44E-05	Other*	Mitochondria*
53577	NA	0.105	0.0285	***	0.0239	Other*	Nucleus*
5896	NA	0.0557	0.0227	15.1	0.0456	Other*	Nucleus*
17293	NA	0.0529	0.0188	***	0.0395	Other	Chloroplast
23739	NA	0.0359	0.0137	***	0.0452	Other*	Nucleus*
6278^#^	Predicted protein	0.0286	0.00995	***	0.0381	Other	Chloroplast
8504	NLR family CARD domain-containing protein 3-like	0.0105	0.0050	2.63	0.0443	Other*	Nucleus*
4654	S8 family serine peptidase	0.00764	0.00155	***	0.0135	Signal peptide (other)	Chloroplast

# indicates proteins also identified as significantly enriched by ConA glycoprotein isolation. NA are sequences with no homologues identified by BLAST. *** are proteins not detected in the total protein sample. * indicates sequences that do not start with a M and so may be truncated sequences that do not contain the targeting sequence present in the protein. Any secretory signal peptides identified were removed and the analysis repeated, with results shown in brackets.

### Extracellular proteome

As well as proteins isolated by lectin-meditated enrichment, the extracellular proteome was analysed. These proteins were isolated from the cell-free media, and it should be noted that a small amount of extracellular media was included in the cell preparation for all other samples. A total of 135 proteins were detected in all three samples of the extracellular proteome, of which 41 were not detected in the total proteome at all. In total, 20 of these were statistically significantly more prevalent than in the total proteome (see Table 4), and of these only two had no BLAST matches (and only one further did not map to a GO term, despite matching a bacterial subtilisin-related peptidase by BLAST). There are several proteins involved in transport and signalling. There is also a lipase, a carbonic anhydrase, a thioredoxin, a peptidyl-prolyl cis–trans isomerase, a glycine dehydrogenase, and interestingly a possible protease inhibitor that could potentially be involved in pathogen resistance (Jashni et al. 2015). There are also several proteins that would not be expected to be extracellular, such as a serine/threonine phosphatase, a chlorophyll-binding protein, and a CoA ligase. Interestingly the most abundant protein, also overrepresented in the ConA samples, does not match any sequences by BLAST.

**Table 4.** Proteins enriched in extracellular proteome.

Sequence name	Putative sequence description	Average intensity	SD	Ratio ext/total	*P*-value	TargetP prediction^23^	WoLF PSORT prediction^24^
2713^+^	NA	72.8	18.1	20200	0.0199	Signal peptide (other)	Extracellular (nuclear)
11740^+^	S8 family peptidase	0.571	0.0849	***	0.00728	Signal peptide (other)	Plasma membrane
13218	Lipase	0.391	0.0583	12400	0.00732	Signal peptide (other)	Extracellular (cytosol)
3168	Long-chain-fatty-acid–CoA ligase 4 isoform X1	0.227	0.0801	2640	0.0392	Other*	Chloroplast*
18460	Hypothetical protein, conserved	0.167	0.0621	***	0.0432	Other*	Chloroplast*
7055	Light-harvesting chlorophyll a/b-binding protein	0.142	0.0495	45.6	0.0392	Other*	Plasma membrane*
1245	P-ATPase family transporter	0.114	0.0307	***	0.0232	Other	Plasma membrane
19656	Cystatin B	0.0959	0.0361	***	0.044	Other	Cytosol
13679	Serine/threonine-protein phosphatase PP-X isozyme 2	0.0851	0.0226	71.5	0.023	Other	Cytosol
14610	Carbonic anhydrase 1	0.0618	0.0196	***	0.032	Other	Chloroplast
6287	CocE/NonD family hydrolase	0.0603	0.0240	292	0.049	Other*	E.R.*
1548	Aminomethyl-transferring glycine dehydrogenase	0.0581	0.0134	12.9	0.023	Mitochondria	Mitochondria
17754	Pleiotropic drug resistance protein ABC superfamily	0.0554	0.0121	480	0.015	Other*	Nucleus*
2873	Probable inorganic phosphate transporter 1–3	0.0476	0.0139	***	0.027	Other*	Plasma membrane*
19178	14–3-3 protein	0.0427	0.00368	7.18	0.0034	Other	Nucleus
13675	3, 5-Cyclic nucleotide phosphodiesterase	0.0342	0.00930	***	0.024	Other	E.R.
1168	ATP-binding cassette subfamily G member 2 isoform X1	0.0337	0.00752	***	0.016	Signal peptide (other)	Plasma membrane
34359^+^	Thioredoxin	0.0286	0.00789	4.02	0.039	Mitochondria	Chloroplast
26116	Peptidyl-prolyl cis–trans isomerase CYP19-3	0.0163	0.00393	5.14	0.034	Mitochondria	Chloroplast
27196	NA	0.0157	0.00128	131	0.0022	Other*	Chloroplast*

NA are sequences with no homologues identified by BLAST. *** are proteins not detected in the total protein sample. Any secretory signal peptides were removed and the analysis repeated, with results shown in brackets. ^+^ indicates deamidation sites that were detected. * indicates sequences that do not start with a M and so may be truncated sequences that do not contain the targeting sequence present in the protein.

## Conclusion

As expected, the most abundant proteins in the total proteome were those associated with core housekeeping roles, central metabolism, and the chloroplasts and mitochondria. Both ConA and WGA were able to enrich for a range of proteins, with some overlap, and the roles some of them may play on the cell surface can be postulated. The extracellular proteome has a number of proteins that could be involved in degrading extracellular material and signalling. A proposed *N*-glycosylation site can be identified in some of the peptides, but it is notable that they are not reliably found at the canonical NX(S/T) sites of other eukaryotes.

Of particular note are the large number of unique proteins, unrelated to any previously identified proteins, that are highly abundant in the total proteome, in the glycoprotein isolation samples, and in the extracellular proteome. These proteins may be truly unique among the Euglena but may also be more widely dispersed among unsequenced organisms. There are also several proteins that are only related to ‘predicted protein’ and with no GO terms identified using Blast2GO. This data indicates there are a large number of highly abundant proteins in Euglena with no known function, some of which we can now tentatively identify as being glycosylated. As well as wider sequencing of diverse eukaryotes to determine their distribution, these unique proteins would repay further biochemical study.

## Supplementary Material

fnac120_Supplemental_Files
